# Multidimensional phenotyping predicts lifespan and quantifies health in *Caenorhabditis elegans*

**DOI:** 10.1371/journal.pcbi.1008002

**Published:** 2020-07-21

**Authors:** Céline N. Martineau, André E. X. Brown, Patrick Laurent

**Affiliations:** 1 Laboratory of Neurophysiology, UNI, Université Libre de Bruxelles, Brussels, Belgium; 2 MRC London Institute of Medical Sciences, London, United Kingdom; 3 Institute of Clinical Sciences, Imperial College London, London, United Kingdom; Washington University in Saint Louis, UNITED STATES

## Abstract

Ageing affects a wide range of phenotypes at all scales, but an objective measure of ageing remains challenging, even in simple model organisms. To measure the ageing process, we characterized the sequence of alterations of multiple phenotypes at organismal scale. Hundreds of morphological, postural, and behavioral features were extracted from high-resolution videos. Out of the 1019 features extracted, 896 are ageing biomarkers, defined as those that show a significant correlation with relative age (age divided by lifespan). We used support vector regression to predict age, remaining life and lifespan of individual *C*. *elegans*. The quality of these predictions (age R^2^ = 0.79; remaining life R^2^ = 0.77; lifespan R^2^ = 0.72) increased with the number of features added to the model, supporting the use of multiple features to quantify ageing. We quantified the rate of ageing as how quickly animals moved through a phenotypic space; we quantified health decline as the slope of the declining predicted remaining life. In both ageing dimensions, we found that short lived-animals aged faster than long-lived animals. In our conditions, for isogenic wild-type worms, the health decline of the individuals was scaled to their lifespan without significant deviation from the average for short- or long-lived animals.

## Author summary

High dimensional biomedical data are used to quantify health and diagnose diseases. Combining the most informative features collected in the best conditions is crucial for predictive power. Using high-resolution videos and extraction of hundreds of morphological, postural and behavioral features, we characterized the phenotypic evolution of worms as they age. Out of the 1019 features extracted, 896 correlate with relative age. We used machine-learning to predict age and lifespan of individual *C*. *elegans* (age R^2^ = 0.79; remaining life R^2^ = 0.77; lifespan R^2^ = 0.72). The quality of these predictions increased with the number of features added sequentially to the model, supporting the use of multiple features to quantify ageing. We evaluated the relationship between ageing and the phenotypic progression. In our conditions, for isogenic wild-type worms, the rate of phenotypic alterations scales with the lifespan of the individuals.

## Introduction

During ageing, multiple phenotypes are altered at the molecular, cellular, tissue and organismal levels. Ultimately, these alterations affect the health and longevity of the organism. The plasticity of ageing was revealed in *C*. *elegans* through several mutations that extend its longevity [1]. Ideally suited for longitudinal studies, the morphology, posture and behavioral repertoire of the worm can be quantified non-invasively. Studies have also used this organism to assess the relationship between healthspan (the fraction of life considered ‘healthy’) and lifespan using phenotypes scored manually, through video tracking, or microscopy [2–7]. The behavior of *C*. *elegans* has been previously shown to change from the first day of adulthood as a consequence of modified neuronal and muscular functions [7–10]. Using locomotion velocity as an indicator of health, lifespan and healthspan were coupled for the long-lived mutant *daf-2* [4,11]. This is in contrast to previous results obtained by Bansal *et al*. [2], showing that long-lived mutants spend a longer period of time in a frail state. Using a set of 5 biomarkers of ageing, Zhang *et al*. showed that long-lived individuals have a longer span of poor health than short-lived wild-type (N2) individuals [6]. Similarly, Podshivalova *et al*. and Churgin *et al*. observed an extension of late-life behavioral quiescence in N2 and *daf-2* mutants [3,5].

Hundreds of morphological, postural and behavioral features can be extracted from videos of freely behaving worms and these high-dimensional phenotypic fingerprints have previously been shown to accurately classify mutants and sensitively detect the effects of optogenetic stimulation [12–14]. We hypothesized that using a higher-dimensional representation of phenotypes would also be useful for quantifying ageing and predicting lifespan. We recorded 151 individuals for 3 minutes each day of their life and used mechanical stimulation to induce locomotion. We identified a set of 896 features that correlate with the age of the worms normalized to their lifespan (relative age), which can be considered biomarkers of ageing. By quantifying changes in these biomarkers, we find that short-lived and long-lived animals age at different rates. In our conditions, the phenotypic and health progression of isogenic wild-type *C*. *elegans* individuals over ageing is simply scaled to their lifespan.

## Results

### Phenotypes correlate better with age and relative age after mechanical stimulation

**Fig 1:**
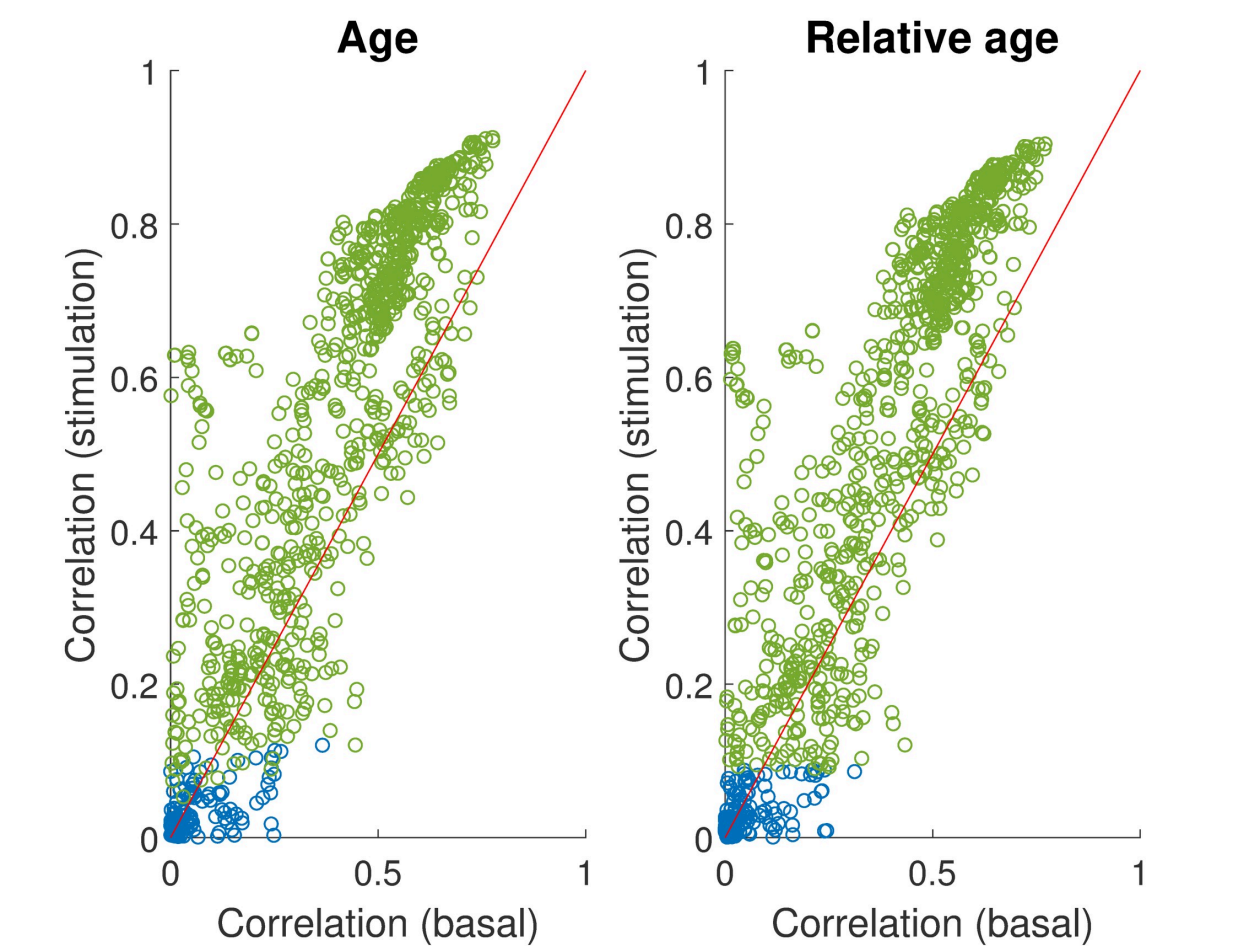
Behavior correlates better with age and relative age following mechanical stimulation. Absolute Spearman’s correlation coefficients of each feature with age and relative age, after mechanical stimulation. Biomarkers of ageing with p values < 0.05 after Bonferroni corrections are depicted in green. Following previous work (e.g. [17]), we define a biomarker of ageing as any phenotype correlating with relative age (fractional age relative to lifespan). To determine which features were the best biomarkers, Spearman’s correlation coefficients with relative age were calculated for each feature after mechanical stimulation. Out of the 1019 features used in this study, 896 had p values < 0.05 after Bonferroni correction. A list of the 100 best biomarkers and their corresponding correlation coefficients is shown in S1 Table. This list includes morphological, postural, and behavioral biomarkers. Many of the Tierpsy features are correlated with each other. Nonetheless, we observed noticeably distinct dynamics between them over ageing (Fig 2). The length of the worm rose during early adulthood and slowly declined afterwards. The maximum speed rose similarly during early adulthood but sharply declined to reach a minimum by mid-adulthood. The curvature at mid-body sharply declined between larval stage 4 and adulthood but remained stable during adulthood. The path-density—a measure of how long the animal dwells in different parts of its path—remains flat in early life but begins to rise around mid-life. To compare short-lived with long-lived animals, our cohort of 151 isogenic animals was split into 5 groups according to longevity. Visualized as a function of relative age, the phenotype changes overlapped between these 5 groups, as expected for biomarkers of ageing.

To assess the health of freely moving animals, we tracked 151 wild-type worms (strain N2 obtained from the *C*. *elegans* Genetics Center) every day of their lives from the last larval stage before adulthood (L4) to death. Mechanical stimulus often triggers a change in direction and fast locomotion. Such high locomotion rates have previously been shown to correlate with remaining lifespan [8,11,15,16]. We therefore recorded worms in basal conditions and after a mechanical stimulation and extracted morphological, postural, and locomotion features using Tierpsy Tracker [12]. For most of the 1019 tested features, Pearson’s and Spearman’s correlation coefficients with age and relative age were higher in stimulated conditions than in basal conditions (Fig 1, S1 Table). The average correlation coefficients with age are 2.25 and 3.48 times higher following stimulation (Pearson and Spearman, respectively); the average correlation coefficients with relative age were 1.98 and 2.92 times higher following stimulation (Pearson and Spearman, respectively). We therefore focused on the features measured following stimulation for the analysis below.

**Fig 2:**
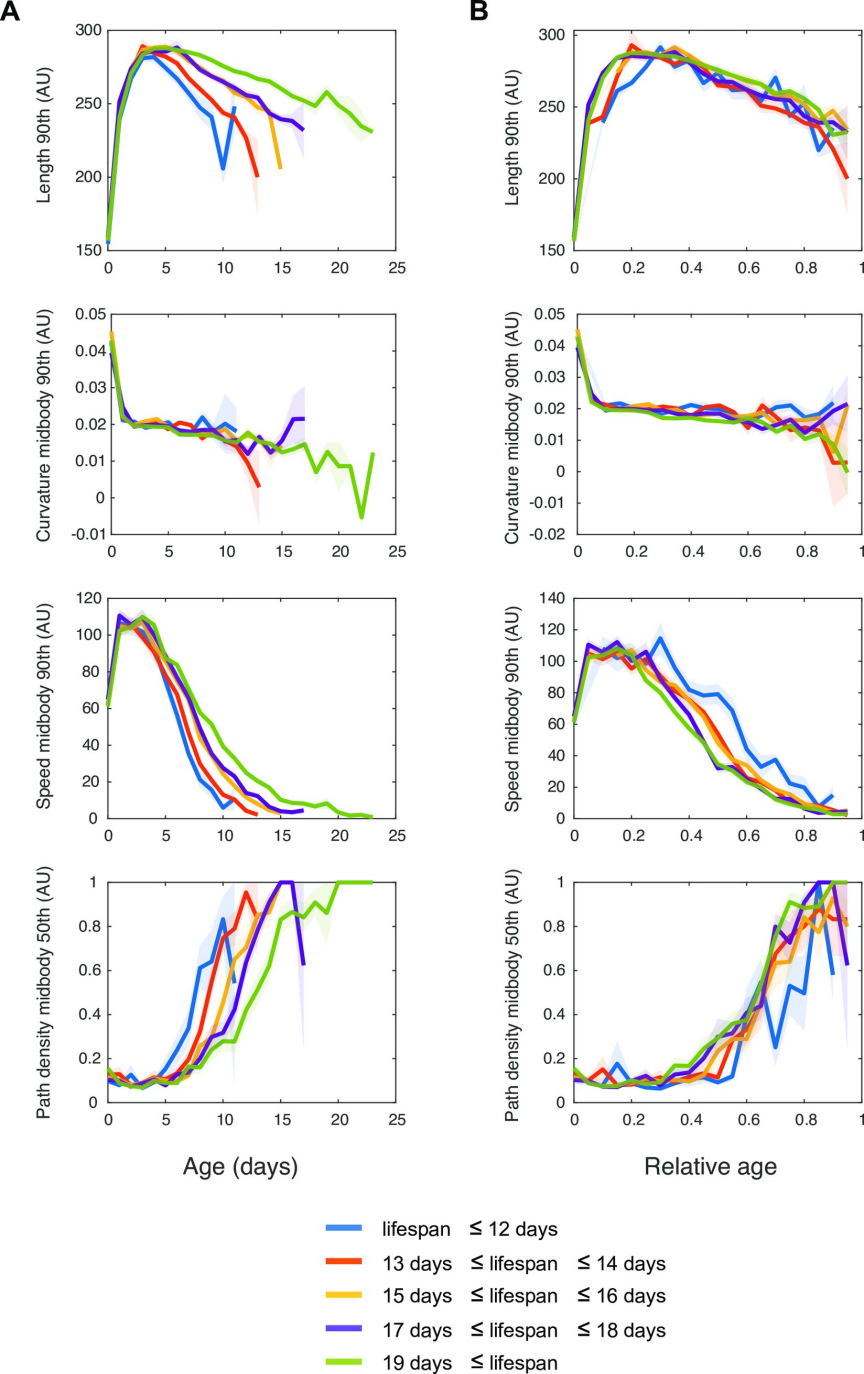
Evolution of 4 features over ageing. The evolution of the maximum (90^th^ percentile) length, maximum speed, and curvature at midbody and the median path density is displayed over chronological age (A) and relative age (B) for 5 groups of longevity. Shaded areas indicate standard errors of the mean.

### Multidimensional phenotyping predicts age, remaining life, and lifespan

**Fig 3:**
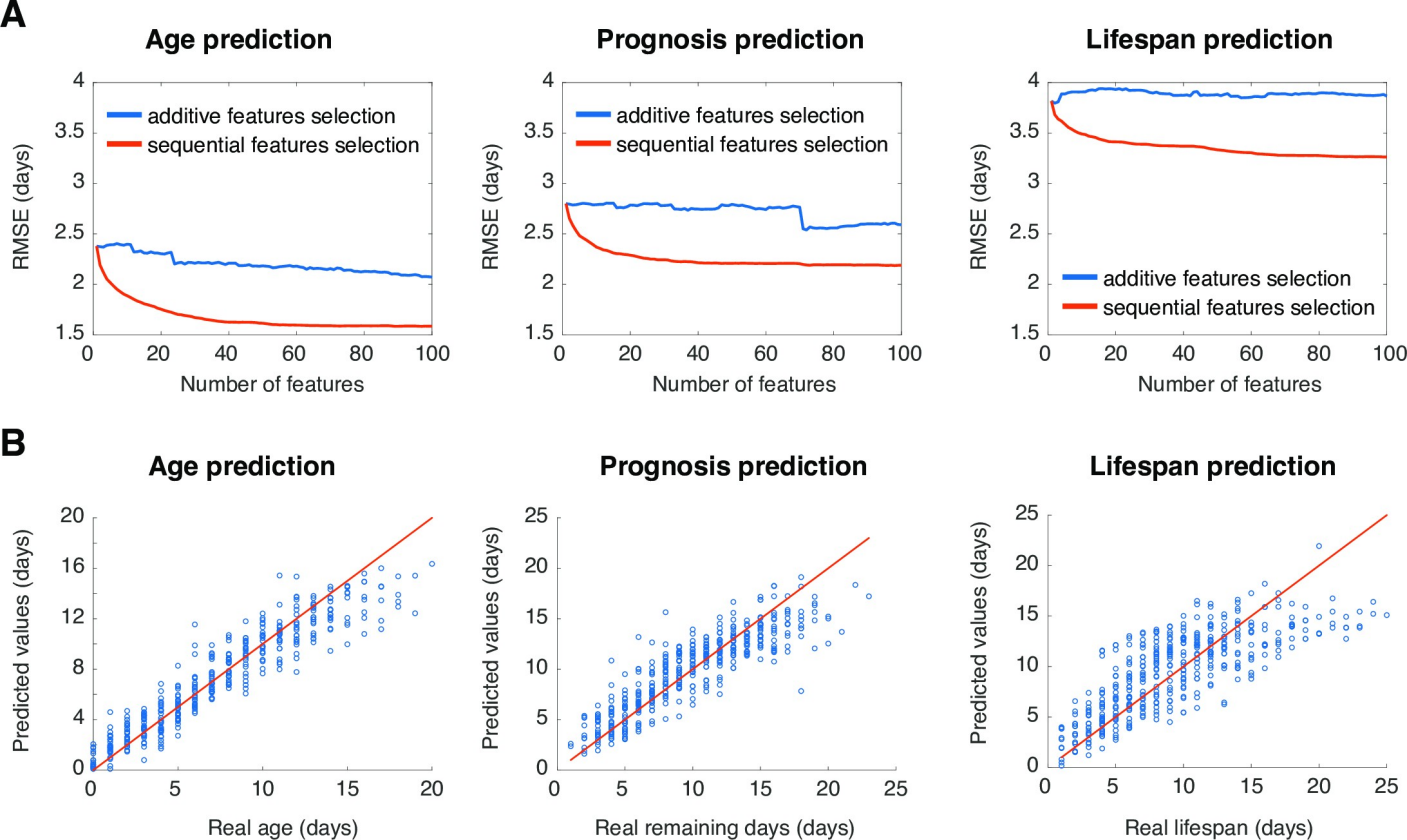
Deep phenotyping predicts age, remaining life and lifespan. (A) Evolution of the Root Mean Squared Error (RMSE) over the number of features added to the SVR models to predict age, remaining life and lifespan. The red line shows the RMSE for features selected using sequential feature selection. The blue line shows the RMSE for features selected accordingly to their ranked prediction error. (B) Prediction of age (R^2^ = 0.79), remaining life (R^2^ = 0.77), and lifespan (R^2^ = 0.72) using 100 features combined iteratively are compared to the real age, remaining life and lifespan. The red line corresponds to perfect predictions.

Given the different ageing dynamics for different features, we hypothesized that they contain independent phenotypic information and could be combined to improve the prediction of age, remaining life, and lifespan. We randomly selected 80% of the individuals as training set and used support vector regression (SVR) to predict age, remaining life, and lifespan of the remaining 20%. We compared two approaches for feature selection. For the additive feature selection approach, we simply ranked features presenting the lowest prediction error and added them one by one to the model. The root mean squared error decreases slowly as features are added, most likely because the highest ranked features are also highly correlated with each other. To select useful features that are also relatively uncorrelated, we also used a sequential feature selection approach. At each iteration, the next selected feature is the one that results in the lowest prediction error when combined with previously selected features. We used a brute force search (testing all remaining features at each iteration) to find the next feature to add. Feature selection was done separately for the prediction of age, remaining life, and lifespan. Using sequential feature selection, the prediction error decreases as more features are added up to approximately 20 to 40 features at which point the accuracy plateaus. The features selected by each method are in S3 Table. From the best set of 100 features, age, remaining life and lifespan were predicted with respective root mean squared errors of 1.6, 2.2 and 3.3 days by the sequential feature selection method (Fig 3A and 3B). For comparison, using all 1019 features led to worse predictions with root mean squared errors of 2.5, 4.5, and 3.8 days for age, remaining life and lifespan predictions, respectively.

### Individuals show progressive phenotypic changes as they age

**Fig 4:**
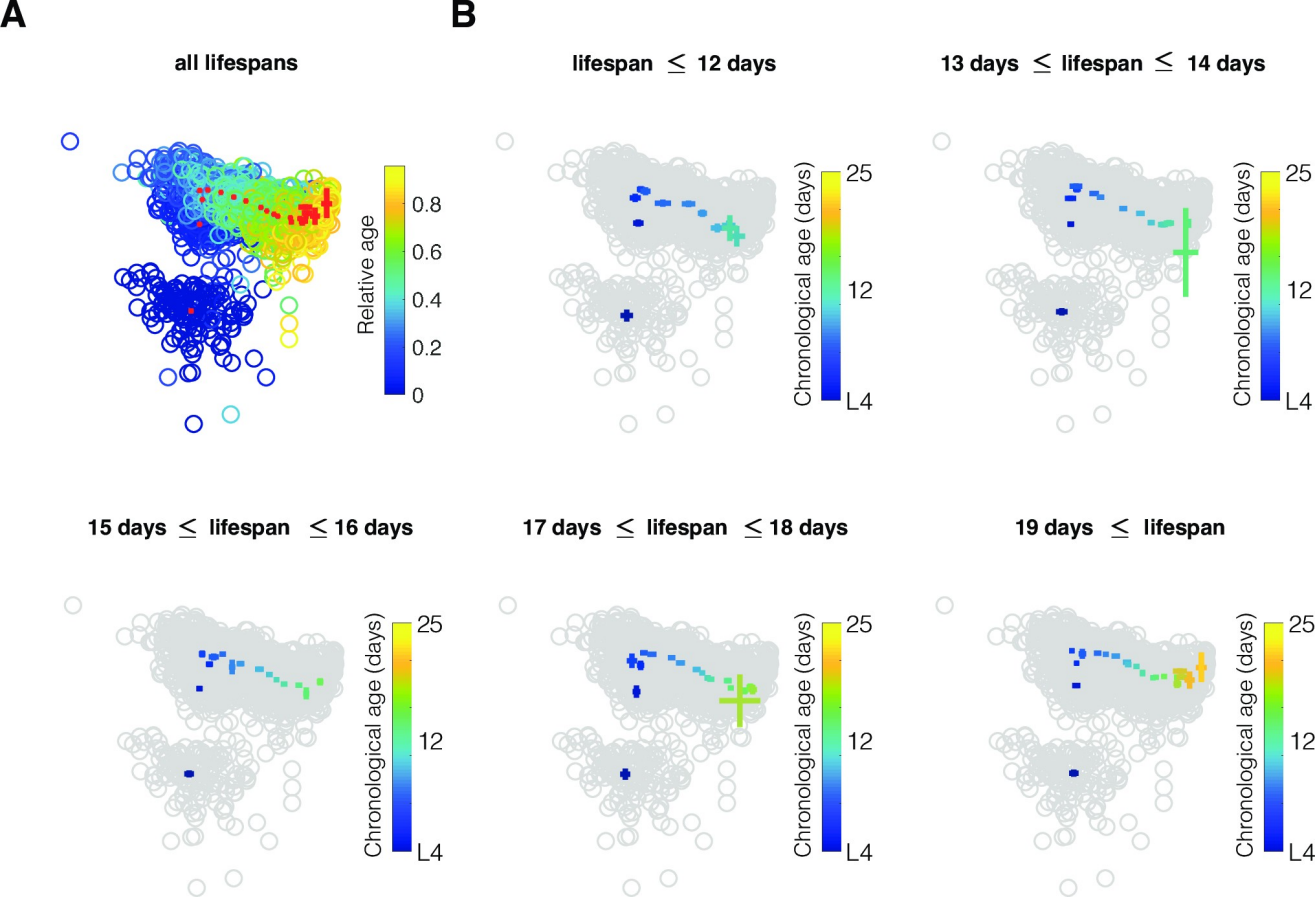
Phenotypic trajectories of ageing wild-type *C*. *elegans*. (A) PCA representation of the phenotypes over age. Each circle corresponds to the phenotype of an individual at a specific age. The colors of the circles indicate the relative age, from L4 (dark blue) to death (yellow). The red crosses indicate the mean phenotype and standard error at each age (B) 5 groups of longevity are compared: the colored crosses indicate the mean phenotype and standard error at each age in the same PCA space as represented in (A). The colors of the crosses indicate the chronological age.

Numerous drugs and genetic manipulations can modulate longevity [1,18]. However, their effects on *C*. *elegans* health progression are often unknown. To visualize the phenotypic progression of our cohort of 151 individuals, we used Principal Component Analysis (PCA) (Fig 4) and t-distributed Stochastic Neighbor Embedding (t-SNE) (S1 Fig) to project the high dimensional phenotypes into two dimensions. In PCA, these first two dimensions represented 49% of the total variance of our dataset and additional dimensions each accounted for less than 10% of the remaining variance (S2 Table). In both PCA and t-SNE, the phenotypes were distributed according to the age of the individuals, generating a relatively well-defined trajectory of ageing in two dimensions. We characterized the phenotypic progression of our cohort split into 5 groups of lifespan. In the subspaces examined, the aging trajectories of the 5 longevity groups appeared qualitatively similar to the ageing trajectory of the full cohort of wild-type animals.

### Short-lived animals age faster than long-lived animals

**Fig 5:**
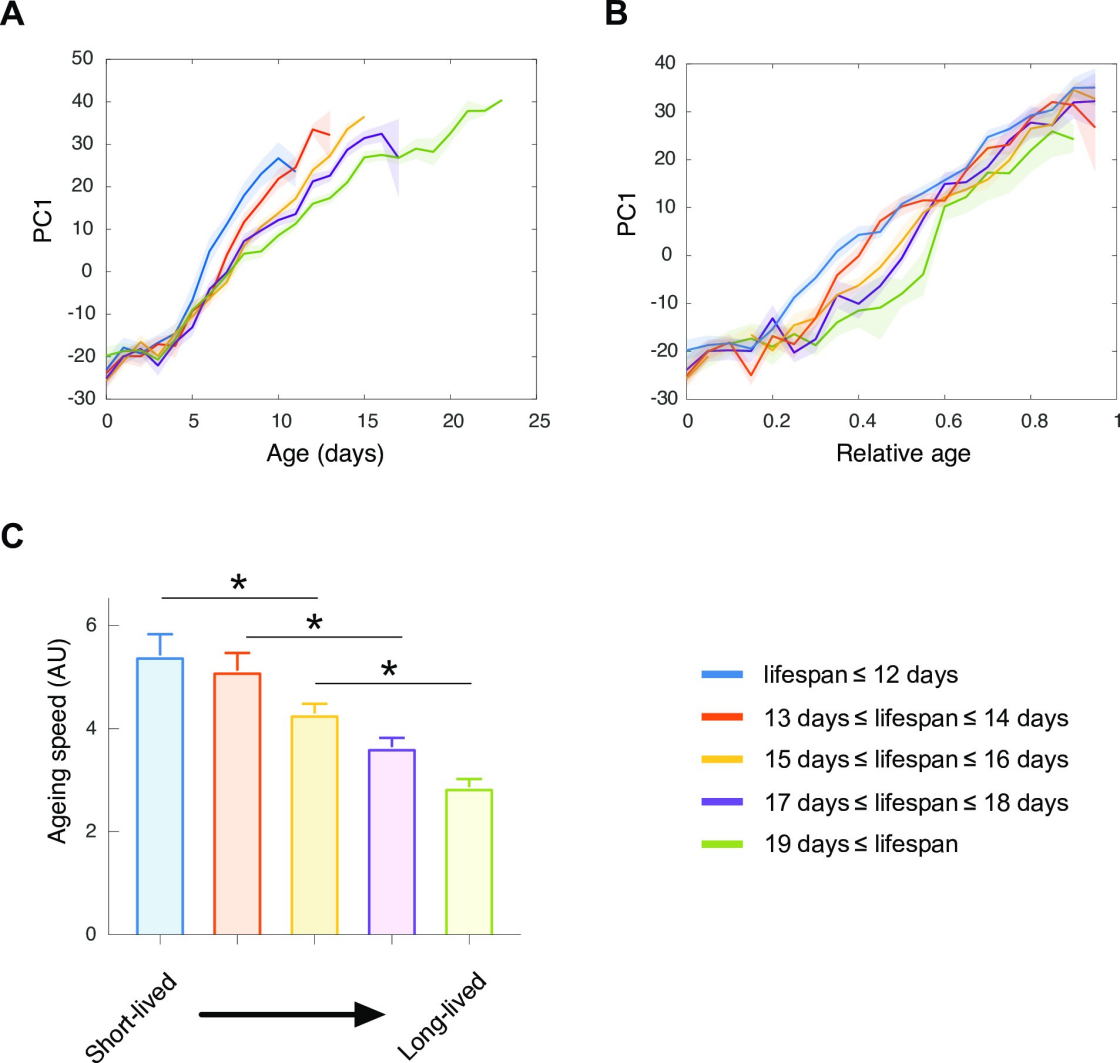
The rate of phenotypic change used as a proxy for the aging rate. The evolution of individuals along the first principal component (PC1) is displayed over chronological (A) and relative (B) age for 5 groups of longevity. The mean for each group of longevity correspond to the colored lines, the shaded areas indicate the standard errors of the mean. (C) The mean ageing rate for each group of longevity was determined as the mean of the first derivative of PC1, error bars indicate the standard errors of the mean.

Interestingly, in both the t-SNE and PCA representations, the short-lived group travelled faster within the phenotypic landscape than the long-lived group. We used the distance between consecutive time points along PC1 as a simple approximation of the phenotypic rate of change. All lifespan groups progress monotonically along PC1 starting soon after the reproductive phase (Fig 5A). However, short-lived groups moved faster than long-lived groups along PC1 (Fig 5C). Visualized as a function of relative age, progression along PC1 is continuous for short-lived animals, it is slightly delayed for long-lived animals (Fig 5B). To confirm that short-lived groups moved faster in the multidimensional phenotypic space than long-lived groups, we computed the cumulated distance travelled by the individuals as they age in higher dimensions, representing from 50% to 90% of the total variance. Short-lived groups travelled faster within the multidimensional phenotypic landscape than long-lived groups (S2 Fig). Therefore, within our multidimensional phenotypic space, the 5 longevity groups display a similar sequence of alterations but at variable rates.

### Long-lived animals have a longer healthspan

**Fig 6:**
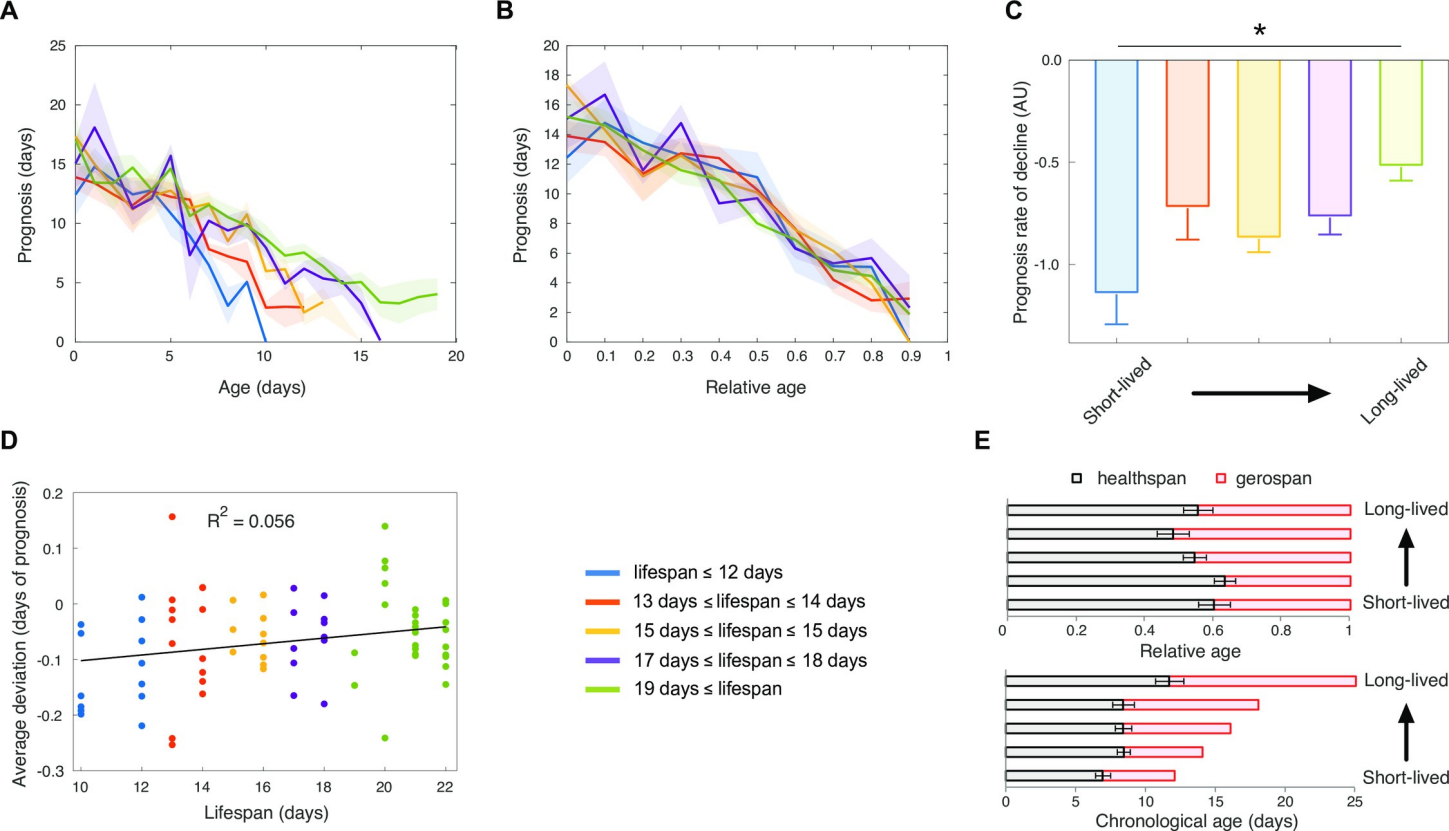
Health progression is coupled to lifespan. (A) Evolution of the predicted prognosis over chronological age. (B) Evolution of the predicted prognosis over relative age. The mean for each group of longevity corresponds to the colored lines, the shaded areas indicate the standard errors of the mean. (C) The prognosis decline slope was determined by fitting a linear curve to the prognosis over chronological age; the error bars indicate the standard errors of the mean. (D) The average prognosis decline was determined for the entire cohort as a reference health decline. The average deviation of the prognosis curve of each individual from this reference health decline was determined and plotted. The R^2^ value is indicated. (E) The healthspan and gerospan were calculated for each group of longevity as the fraction of life above 50% of the initial predicted prognosis value; the error bars indicate the standard errors of the mean.

To more rigorously assess the animals’ health, we used the prognosis, which corresponds to the remaining time until death predicted based on an animal’s current phenotype, as previously defined by Zhang *et al*. [6]. Consistent with previous work, we observed that prognosis decreases with age and decreases more rapidly for worms having a short lifespan (Fig 6A and 6C). However, over relative age, the evolution of prognosis was similar for the different lifespan groups (Fig 6B). The fraction of lifespan in good health was suggested to be extended in short-lived and reduced in long-lived animals compared to the average [6]. To check for the same effect in our dataset, we calculated the deviation of the prognosis curve of each individual from the average prognosis curve of the entire cohort. Positive deviation results from worms spending more time healthier than average while negative deviation results from worms spending more time being less healthy than average. We did not detect a significant deviation from the reference prognosis decline in long-lived or short-lived worms. This result suggests that health decline normalized to lifespan is similar for all longevity groups in our conditions (Fig 6D). From the prognosis, we calculated healthspan as the fraction of life above 50% of the initial predicted prognosis value; the gerospan corresponds to the fraction of life below this threshold. Based on this healthspan and gerospan criterion, the fraction of lifespan in good and bad health appears similar for long and short-lived cohorts (Fig 6E).

## Discussion

*Caenorhabditis elegans* exhibits many age-associated changes in gene expression, protein quality control systems, tissue integrity and immunity [19]. Individual features such as *hsp-16*.*2* expression, pharyngeal pumping rate, maximum velocity (at day 9 of adulthood) or a combination of multiple features such as movement, auto-fluorescence, and body size were previously shown to correlate well with lifespan or to predict remaining life [4,6,11,20]. We used multidimensional phenotypes derived from worm tracking to attempt to access more of these non-invasive biomarkers. We found that sequential feature selection improved lifespan prediction compared to simply taking the most predictive features. Adding more selected features improved predictions until the support vector regression model used approximately 20 to 40 features, accordingly to predictions, at which point their accuracy plateaued.

Our results show that phenotypes evoked by mechanical stimulation, including features measuring the maximum locomotion capacities of the worms, better correlate with age and relative age than their counterparts obtained in basal conditions. This is consistent with previous results showing that the maximum velocity of the animals correlates better with lifespan than the mean velocity [3,4,16]. This approach is similar to the Short Physical Performance Battery (SPPB), which combines standing balance, walking speed, and chair stand tests to evaluate maximal physical performances of human patients and their biological age. This similarity supports analogies in the ageing processes between species despite different morphologies, postures, and locomotory phenotypes [21–23].

Detailed phenotypic trajectories of ageing better represent the multifaceted responses to perturbations happening over ageing than lifespan curves. Qualitatively, the short- and long-lived wild type animals followed similar phenotypic trajectories in the subspaces we selected. Using the same methodology, we previously observed that different genotypes can follow distinct trajectories [24].

In our conditions, the inter-individual differences in phenotype are mostly explained by a scaling of the phenotypic progression accordingly to the lifespan of the individuals. This observation is reminiscent of the temporal scaling observed for lifespan curves [25]. Similarly to [2,4,6], we observe that health decline and phenotypic progression occur at different rates in short- and long-lived wild type animals. However, normalized to the lifespan, the dynamics of health decline for short- and long-lived wild-type animals could not be distinguished. This result differs from [6] but is consistent with other previously published results [4].

The features selected to measure health, the environment and/or the genotype used may explain these discrepancies between studies. We explore more features than previous publications [2,4,6] although using fewer individuals than in [6]. Our features are mainly morphological and locomotory, lacking indicators for reproduction or tissue integrity present in [6], which might be crucial markers for health quantification. We used N2, while [6] used the sterile strain *spe-9(hc88)*. We transferred individual worms every day onto fresh bacteria with an eyelash, maintaining the environment relatively constant at the cost of worm handling. In contrast, [6] maintained individual worms over their entire life in droplets of concentrated bacteria sealed in hydrogel and observed “premature death” in short-lived worms as well as “extended twilight” for long-lived worms. In this closed environment, declining bacterial concentrations are likely coupled with increasing metabolite concentrations. Low concentrations of bacteria might increase longevity and the frail end of life by reducing mortality induced by bacterial infection [5,26]. Evolving bacterial densities might therefore explain both the “premature death” of short-lived worms living in a high density of bacteria as well as the “extended twilight” of long-lived worm living in a reduced density. In addition, small molecules produced by bacteria or by worms can accumulate in the droplet, potentially modulating the longevity of the worm [27–29].

In conclusion, the sequence of alterations of multiple organismal phenotypes is highly predictive of age, lifespan and prognosis of individual *C*. *elegans*. In our conditions, for isogenic wild-type worms, the rate of phenotypic alterations scales with the lifespan of the individuals.

## Materials and methods

### Strains and media

The wild-type strain N2 (obtained from the *C*. *elegans* Genetics Center) was used in this study. Standard conditions were used to maintain and propagate this strain at 20°C.

### Collection of behavioral data

151 single worms were recorded using a worm tracker equipped with a bone conductor transducer for mechanical stimulation. Worms were tracked longitudinally every day from the L4 stage to death. Worms were maintained in strict conditions at 20°C until and during the tracking. Single-worm tracking was performed as previously described [14] with slight modifications. Briefly, 3 cm plates containing low peptone NGM were seeded with 20 μL of OP50 30 minutes prior tracking. Each day, each single worm was picked with a sterile eyelash onto a new freshly seeded plate and left to acclimate for 15 minutes. All animals were measured within a period of 3 months, but not all animals were followed at the same time. After 2 minutes of acclimation in the tracker, worm behavior was recorded for 2 minutes at 20 frames per second to extract basal phenotypes. To extract phenotypes after mechanical stimulation, behavior was recorded for 5 seconds in basal condition before a vibration of 4 seconds at 750 Hz was applied, and recorded 1 more minute after stimulation.

### Data preparation

Videos were analyzed using Tierpsy to extract behavioral features [12]. Tierpsy is freely available at https://github.com/Tierpsy/tierpsy-tracker. The extracted worm behavior dataset as well as information for each feature are available on https://github.com/celegans-ulb/MultidimensionalPhenotyping. For each worm, a set of 4539 features was extracted with the Tierpsy software [12]. These features contain information about the worm morphology, posture, locomotion, and behavior. After feature extraction, worms containing more than 25% of missing values were removed from the dataset. Features still containing missing values were dropped for all the videos. The dataset was then z-normalized to allow comparison across features with different units.

### Correlation p values

For each feature, Pearson's and Spearman's correlation coefficients (and the associated p-values) with age and relative age were calculated using the default settings in the MATLAB statistics toolbox.

### Predictions

Predictions were performed using support vector regression with a linear kernel using the fitrsvm and predict functions in MATLAB. The code used is available on https://github.com/celegans-ulb/MultidimensionalPhenotyping. The training set comprises 80% of the individual animals randomly selected from the total. Predictions were made on the remaining 20% of animals and compared to their actual age, remaining life, and lifespan. The training set includes the phenotypes as predictor values and the corresponding age, remaining lifespan or lifespan as response value. Predictions were compared to the real age, remaining lifespan or lifespan. Lifespan was predicted from prognosis, to which the actual age of the animal was added. To measure prediction accuracy, we used the root mean squared error (RMSE) of the prediction. To plot the prediction precision over the number of features, features were added to the model one by one. For the sequential feature selection method, the feature giving the smallest root mean squared error was selected at each iteration. All remaining features were tested at each iteration to find the next best feature to add. For the additive feature selection method the features were selected one by one according to their ranked individual RMSE. Feature selection was done separately for the prediction of age, remaining life, and lifespan.

### Cumulative distance as a measure of ageing

To quantify the change in phenotypes over time for different longevity groups, we calculated the cumulative distance that each group travelled through phenotype space. The space was defined as the first n principal components calculated using all individuals at all times. Each longevity group was represented by a single point in this phenotype space which was the average of the individual worm phenotypes in the group on each day. The distance travelled at each day is the Euclidean distance between the mean phenotype at the current day and the mean phenotype on the previous day. The cumulative distance travelled is then the cumulative sum of these distances over time. The phenotypic distance travelled was calculated independently using principal components 1 to 2, 1 to 5, 1 to 12, 1 to 28, and 1 to 72.

### Prognosis as a measure of health

Prognosis was predicted as described above and plotted over age or relative age. Average deviation of prognosis was calculated as in [6]. If the level of health decreased uniformly throughout life, plotted in relative age, it should follow a straight-line from the initial prognosis to the prognosis at death. We calculated a neutral straight line corresponding to the population mean starting prognosis to the mean prognosis at death. Individuals might have a positive or negative deviation from the neutral, straight-line. The total deviation is calculated as the area between the actual trajectory of prognosis and a neutral straight line corresponding to the population mean starting prognosis to the mean prognosis at death. For each individual, the total area is divided by the lifespan to obtain the average deviation. Healthspan was determined as the time when 50% of initial prognosis was reached. Gerospan corresponds to the remaining time of life (healthspan subtracted to lifespan).

## Supporting information

S1 Fig Phenotypic progression of the 5 lifespan groups. t-SNE representation of the phenotypes over age for 5 different lifespan groups. Colour of each dot indicates the relative age, from L4 (dark blue) to death (yellow). Crosses indicate mean phenotype and standard error of the mean for each age. The black crosses indicate the mean phenotype and standard error for the entire dataset at each age. The red crosses indicate the mean phenotype and standard error at each age for a given lifespan group. (A) PCA using only the stimulated features, (B) PCA using basal and stimulated features.
(TIF)

S2 Fig Short-lived animals age faster than long-lived animals. (A, B) The evolution of individuals along the first principal component (PC1) for stimulated features (A) or all features (B) is displayed over chronological and relative age for 5 groups of longevity. The mean for each group of longevity correspond to the coloured lines, the shaded areas indicate the standard errors of the mean. (C) For stimulated features, the evolution of the cumulative distances of individuals across multiple dimensions over chronological age for 5 groups of longevity (colours as in A and B). (D) Ageing speed across multiple dimensions.
(TIF)

S1 Table Biomarkers of ageing. Spearman’s correlation coefficients with relative age were calculated for each feature after mechanical stimulation.
(XLSX)

S2 Table Percentage of variance explained by the 10 first principal components for features after mechanical stimulation. (XLSX)

S3 Table Features selected by the additive feature selection and sequential feature selection methods to predict age, remaining lifespan and lifespan. (XLSX)

S4 Table Features better correlated in basal conditions than stimulated conditions. (XLSX)
