# Supplementary figures and images for: Multidimensional phenotyping predicts lifespan and quantifies health in Caenorhabditis elegans
Source: PLoS Comput Biol. 2020 Jul 21;16(7):e1008002. doi: 10.1371/journal.pcbi.1008002 (PMC7394451; doi:10.1371/journal.pcbi.1008002)

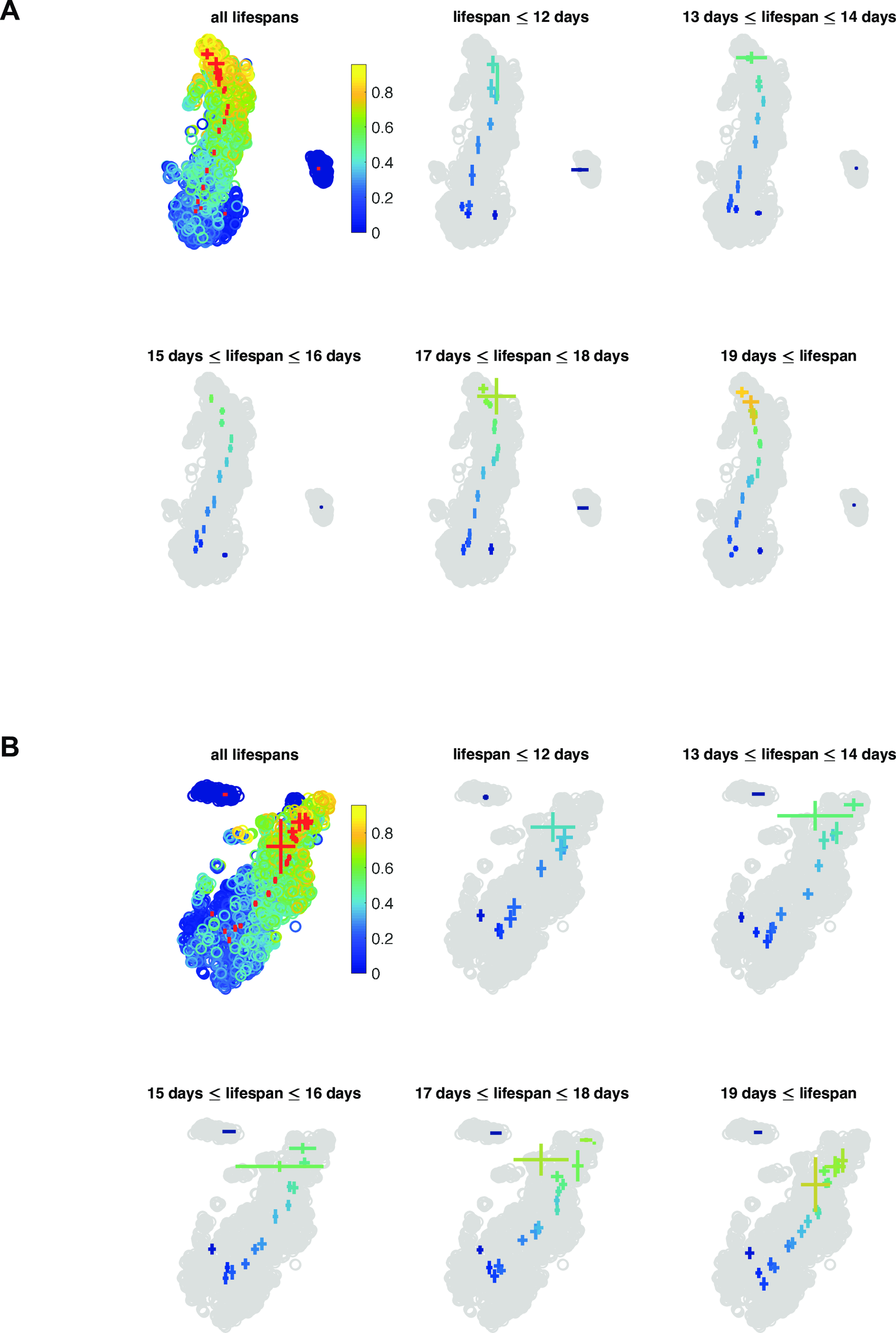

Supplement: S1 Fig — t-SNE representation of the phenotypes over age for 5 different lifespan groups. Colour of each dot indicates the relative age, from L4 (dark blue) to death (yellow). Crosses indicate mean phenotype and standard error of the mean for each age. The black crosses indicate the mean phenotype and standard error for the entire dataset at each age. The red crosses indicate the mean phenotype and standard error at each age for a given lifespan group. (A) PCA using only the stimulated features, (B) PCA using basal and stimulated features. (TIF) [file pcbi.1008002.s001.tif]

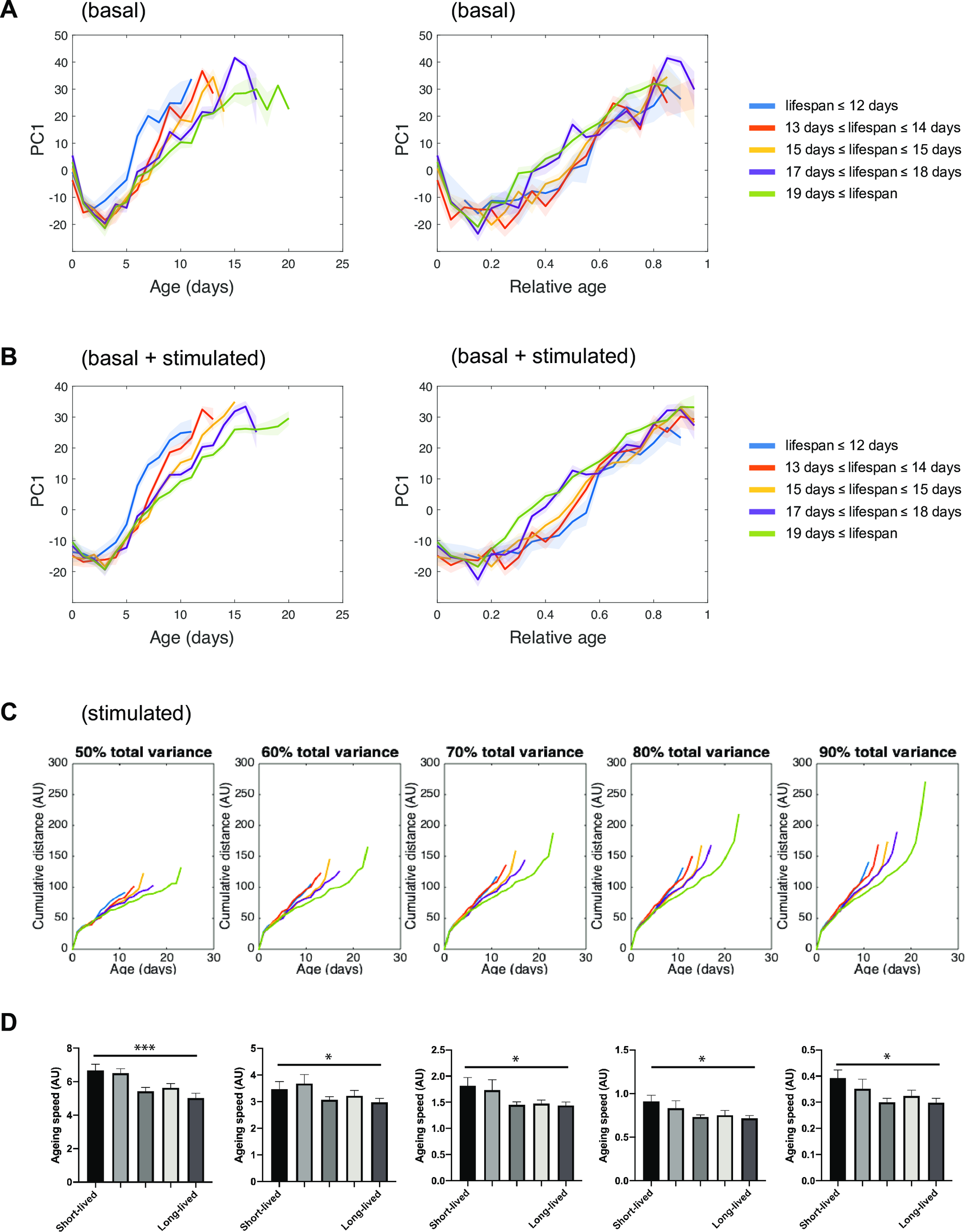

Supplement: S2 Fig — (A, B) The evolution of individuals along the first principal component (PC1) for stimulated features (A) or all features (B) is displayed over chronological and relative age for 5 groups of longevity. The mean for each group of longevity correspond to the coloured lines, the shaded areas indicate the standard errors of the mean. (C) For stimulated features, the evolution of the cumulative distances of individuals across multiple dimensions over chronological age for 5 groups of longevity (colours as in A and B). (D) Ageing speed across multiple dimensions. (TIF) [file pcbi.1008002.s002.tif]
